# Attachment Style and Chronic Pain: Toward an Interpersonal Model of Pain

**DOI:** 10.3389/fpsyg.2017.00284

**Published:** 2017-02-24

**Authors:** Annunziata Romeo, Valentina Tesio, Gianluca Castelnuovo, Lorys Castelli

**Affiliations:** ^1^Department of Psychology, University of TurinTurin, Italy; ^2^Psychology Research Laboratory, IRCCS Istituto Auxologico ItalianoVerbania, Italy; ^3^Department of Psychology, Catholic University of MilanMilan, Italy

**Keywords:** attachment style, chronic pain, coping, catastrophizing, insecure attachment

## Abstract

Chronic pain (CP) is a burdensome symptom. Different psychological models have been proposed to explain the role of psychological and social factors in developing and maintaining CP. Attachment, for example, is a psychological construct of possible relevance in CP. The first studies on the role of attachment in CP did not investigate the partner’s psychological factors, thus neglecting the influence of the latter. The main aim of this mini-review was to examine the more recent literature investigating the relationship between CP and attachment style. In particular, whether or not more recent studies assessed the psychological variables of a patient’s partner. The articles were selected from the Medline/PubMed database using the search terms “attachment” AND “pain”; “CP” AND “attachment style,” which led to nine papers being identified. The results showed that, even though the key point was still the hypothesis that an insecure attachment style is associated with CP, in recent years researchers have focused on the possible psychological aspects mediating between attachment style and CP. In particular, worrying, coping strategies, catastrophizing and perceived spouse responses to pain behavior were taken into account. Only one study considered the role of the reciprocal influence of attachment style of both patient and partner, underlining the role of real significant others’ responses to pain behaviors. In conclusion, the results of the present mini-review highlight how in recent years researchers have moved toward investigating those psychological aspects that could mediate the relationship between attachment and CP, while only partially evaluating the interpersonal perspective.

## Pain and Psychological Theories

Pain is a burdensome symptom, which not only doctors, but also mental health specialists find themselves facing in patients.

The International Association for the Study of Pain (IASP) defined pain as “an unpleasant sensation and an emotional experience associated with a real or potential damage to tissue, or the equivalent of such damage” (Merskey and Bogduk, 1994). To better reflect the interconnection between physical and psychological sensations in pain experience, the fifth edition of DSM (American Psychiatric Association, 2013) replaces somatoform disorders with somatic symptom and related disorders.

Pain is traditionally divided into acute pain and chronic pain (CP). The function of acute pain is to alert the subject and motivate action in order to avoid tissue damage; while CP is defined as pain lasting for at least 3 months. Many factors (neurobiological, psychological, and social) can contribute to pain continuing over time (Nesse and Ellsworth, 2009).

Chronic pain can involve several biological processes, including joint degeneration, inflammation (e.g., rheumatoid arthritis), tumor growth (cancer pain), damaged nerves (neuropathic pain) and can affect different body locations. CP could also be present in multi-symptom syndromes, including, for example, Fibromyalgia (FM) or irritable bowel syndrome (Lumley et al., 2011). These syndromes are also called *Central Sensitivity Syndrome* (CSS). Symptom severity in CSS is often underrated, because patients’ complaints are considered as “all in their heads,” a phenomenon better described with the term somatization – the tendency to experience, communicate, and seek care for somatic symptoms that are disproportionate to pathological findings (Lipowski, 1988).

The relationships between CP and psychological aspects are quite articulated and complex (Castelnuovo et al., 2016a,b). The literature proposes different psychological models of CP to explain the role played by psychological and social factors in developing and maintaining CP.

Emotional regulation skills are often taken into account. With the *Neuroscience Model of Alexithymia*, Lane et al. (2009) suggested that the limited ability for emotional awareness and verbalization of CP patients with alexithymia may lead them to describe the physiological aspects of emotions in somatic terms (Lumley et al., 2007).

The *Fear-Avoidance Model of Chronic Pain* (Asmundson et al., 1999; Norton and Asmundson, 2004) hypothesized that anxiety amplifies the intensity of emotional reactions and the tendency to avoid activities, both of which increase the risk of maintaining CP. According to this model, catastrophic thinking and the fear of movement lead to the maintenance of fear and hypervigilance in relation to bodily sensations. What is more, Cano et al. (2000, 2004) found that high-catastrophizing patients might express their needs for support in aversive ways, which may cause family members to react negatively.

Evidence from recent studies suggests that attachment is another psychological and social construct that could play a relevant role in pain experience. The Attachment Theory was conceptualized by Bowlby (1969) to give a biological framework to psychological development and to explain close and care relationships. An attachment relationship is a strong emotional bond that ideally ensures support and care in case of illness or threat, and ideally gives a sense of security and safety. According to Bowlby’s (1969) theory, children, over time, internalize experiences with care-takers in such a way that early attachment relations come to form a prototype for later relationships outside the family. The models of the self and the other, as conceptualized by Bowlby, can be combined to describe prototypic forms of adult attachment. The model of the self, which is the individual’s perception of the self, determines the extent to which individuals consider themselves worthy of support and proximity; the model of others represents the individual’s perception of others and affects the individual’s confidence of receiving support from them.

The combination of these models yields a classification of one secure and three insecure adult attachment styles: preoccupied, fearful-avoidant, and dismissive-avoidant. Secure attachment is characterized by a sense of worthiness (lovability) combined with an expectation that other people are generally accepting and responsive. Preoccupied attachment refers to a sense of unworthiness (unlovability) combined with a positive evaluation of others. Fearful-avoidant attachment is characterized by a sense of unworthiness (unlovability) combined with an expectation that others will be negatively disposed (untrustworthy and rejecting). Finally, dismissive-avoidant refers to a sense of love-worthiness combined with a negative disposition toward others (Bartholomew and Horowitz, 1991).

Based on the attachment theory, different studies have tried to investigate how mental representations of attachment relationships may influence physiological responses, health behavior, affective states, and health outcomes (Pietromonaco et al., 2013). Maunder and Hunter (2001) underlined the hypothesis that insecure attachment can contribute to physical illness by means of three mechanisms: altered stress physiology, increase use of external regulators of affect, and altered use of health-protective behaviors. As a predictor of stress-vulnerability, insecure attachment may therefore be considered a risk factor for different diseases, including CP.

### Evidence and First Theories Using Attachment Style as a Framework to Explain Chronic Pain Behaviors

Different studies have proposed alternative models for attachment in CP, but all underlined the role of insecure attachment as the greatest risk factor for developing CP (Mikail et al., 1994). The earliest model using attachment theory to explain pain behaviors was theorized by Kolb (1982), and suggested a specific insecure attachment pattern in individuals with CP. Characterized by clinging, complaining, impulsivity, a high level of depression, anxiety and help seeking, this attachment pattern was the result of a lack of a secure base in childhood.

Meredith et al. (2008) reviewed the evidence linking adult attachment theory and CP, and developed the *Attachment Diathesis Model of Chronic Pain*, which underlines the relationship and reciprocal influence between pain and attachment. Their review concluded that insecure attachment represents a predisposition to developing CP, and correlates with different psychosocial variables implicated in pain: insecurely attached people reported more depressive symptoms, maladaptive coping strategies and lower pain self-efficacy than those with secure attachment. In particular, anxious attachment is linked to the fear and/or conviction of having a serious disease. What is more, anxiously attached individuals tend to catastrophize their pain and emphasize their negative feelings to elicit more support from others (Meredith et al., 2008).

It should be noted that these first studies did not investigate the partner’s psychological factors. Indeed, most of these studies on attachment and pain took into account an intrapersonal perspective to analyze pain variables, neglecting the influence that a partner’s psychological factors could have on the pain and psychological health of the patient.

On these bases, the main aim of this mini-review was to examine the literature published after the review of Meredith et al. (2008), which had, using the attachment theory as a theoretical framework, investigated the relationship between CP experience and attachment. In particular, in order to give a more complex and exhaustive framework to this topic, we aimed to investigate whether more recent studies have assessed the psychological variables of a patient’s partner.

### Methods

To analyze the link between adult attachment style and pain-related variables, a search for recent (from 2008 to date) literature on this subject, focusing in particular on CP conditions, was conducted on the Medline/PubMed database using the following terms: “attachment” AND “pain”; “CP” AND “attachment style.” The inclusion criteria for target papers were: CP patients, adult attachment style as a predictor variable, pain constructs as the outcome variables, and being written in English.

### Results

Using the inclusion criteria, nine papers were identified and included in the present review (**Table 1**). Studying chronic widespread pain condition (CWP), Davies et al. (2009) found that attachment style did not in fact directly affect pain intensity, but that patients with insecure attachment presented higher levels of pain disability and more pain sites compared to patients with secure attachment. The lack of a direct relationship between attachment dimensions and pain duration was underlined in another study by Andersen et al. (2011), which investigated attachment as a vulnerability factor in the relationship between chronic whiplash associated disorder (CWAD) and post-traumatic stress disorders (PTSD). Variables such as traumatic events, sensory symptoms, and cognitive symptoms were taken into account. The results underlined that there were no statistically significant correlations between attachment dimensions and pain, but that anxious attachment was moderately correlated with both somatization and PTSD symptoms.

**Table 1:** Studies about attachment style and chronic pain.

Authors	Sample	*N*	Attachment measures	Pain variables	Covariates	Main results
Oliveira and Costa, 2009	Women with FM.	128	RAQ	Pain coping.	Health status, sociodemographic information.	Worrying was correlated with dependence and ambivalence, mediated the relationship between dependence and physical health status, and partially mediated the relationship between ambivalence and mental health status.
Davies et al., 2009	462 adults with CWP; 1041 with other pain; 1006 pain free.	2509	RQ	Pain intensity, chronicity of pain symptoms; sites of pain.	Demographic information.	Individuals with CWP were 2.6 times more likely to report a preoccupied attachment than those free of pain; CWP subjects with a preoccupied attachment were more likely to report a high level of pain-related disability than those with a secure attachment.
Andersen et al., 2011	Individuals with CWAD.	1618	RAAS	Pain symptoms, sensory symptoms; painful episodes.	Symptomatology and severity of PTSD, distress symptoms.	High prevalence of insecure attachment; there was a moderate correlation between anxious attachment and somatization and with PTSD symptoms.
Andersen, 2012	Patients with a diagnosis of non-malignant CP condition.	72	RAAS	Pain intensity; pain disability.	Depressive and anxiety symptoms; use of medication.	The insecurely attached group was significantly more anxious and depressed at both pre- and post-treatment compared to the securely attached group; none of the attachment dimensions was associated with opioids, pain intensity, or physical disability.
Forsythe et al., 2012	Persons (male and female) with CP.	182	RSQ; RQ	Pain intensity; pain behavior; perceptions of spouse responses to pain behaviors.	Disability; depression.	Preoccupied and fearful attachment styles were positively associated with self-reported pain behaviors and intensity. Perceived negative spouse responses partially mediated the relationship between preoccupied attachment and self-reported pain behavior.
Kratz et al., 2012	Women with CP and diagnosis of osteoarthritis.	210	RQ	Pain intensity; pain catastrophizing; pain coping.		Avoidant individuals endorsed higher mean pain and pain catastrophizing compared to those low in avoidance. Avoidant individuals also endorsed significantly lower mean social coping compared to those low in avoidance.
Kowal et al., 2012	Patients with tertiary CP.	238	ECR-R	Pain intensity; pain self-efficacy.	Demographic information; depression; self-perceived burden; caregiver burden.	People with CP, with a high level of anxious attachment may be more demanding toward their caregiver, which may contribute to caregiver burden as well as feelings of SPB in the care receiver.
Gauthier et al., 2012	Patient with advanced cancer.	191	ECR-R	Pain severity; pain catastrophizing; caregiver responses.	Cognitive variables; physical functioning.	Greater catastrophizing was related to more frequently perceived distracting and solicitous responses; higher pain catastrophizing was associated with less frequent punishing responses only among anxiously attached patients.
Monin et al., 2014	Individuals with a self-reported musculoskeletal condition and their spouses.	154	ECR-R	Average pain; pain medication.	Depressive symptoms; marital satisfaction; perceived social support; physical conditions.	When both partners had high anxious attachment, the one with the musculoskeletal condition reported the greatest depressive symptoms; a high level of avoidance attachment was associated with lower marital satisfaction for both partners.

Another study (Andersen, 2012) pointed out that neither avoidant attachment nor anxious attachment were significantly and directly related to pain intensity. Nevertheless, patients with avoidant attachment presented a high level of physical disability, while patients with anxious attachment tended to present a high level of psychosocial disability (Andersen, 2012).

All these studies suggested that there is no direct relationship between pain intensity and attachment, and that this relationship is more probably mediated by other psychological factors.

Although the hypothesis of an association between insecure attachment style and CP remained the key point, in fact, in recent years researchers have focused on the possible psychological aspects that mediate between attachment style and CP. Variables like marital satisfaction, mood, health status, coping strategy, and cognitive dimensions have recently been investigated as mediators of the influence of attachment style on CP conditions.

Oliveira and Costa (2009) investigated the associations in FM between the adult attachment dimensions that they defined as trust, dependence, avoidance and ambivalence, perceived health status and worrying. The results showed an intercorrelation between attachment dimensions and worrying (i.e., anxiety over health status), mental and physical health in patients with FM. In particular, worrying mediated the relationships between dependence and both physical and mental health status, and partially mediated the relationship between ambivalence and mental health status (Oliveira and Costa, 2009).

The coping strategies represent another psychological factor that has been evaluated as mediator. Patients with CP generally show a high level of anxiety, resulting in an exaggerated response to stress and a consequent tendency to catastrophize pain.

Kratz et al. (2012) analyzed attachment pattern as a predictor of daily catastrophizing and social coping in women with osteoarthritis and FM, through a daily diary methodology. As expected, the findings indicated that women with anxious attachment and a relatively poor concept of self-reported a greater increase in catastrophizing during the days of major pain compared to women with a non-anxious attachment style (Kratz et al., 2012). Furthermore, and in agreement with the authors’ hypothesis, women with higher avoidant attachment manifested a greater reticence to cope socially compared to women with lower avoidant attachment (Kratz et al., 2012). Taken together, the data showed that, through its influence on the coping strategies, including catastrophizing, attachment style has an indirect effect on pain management.

Although the above-mentioned studies investigated a wider range of variables related to the pain experience (catastrophizing, coping style) they did not examine the influence of attachment and catastrophizing in the relationship between the patient’s help-seeking behaviors and the partner’s support provision. Gauthier et al. (2012) proposed the first study of the roles of pain catastrophizing and attachment style in cancer patients, using the *Communal Coping Modeling* (CCM) of catastrophizing. According to the CCM, catastrophizing could have the interpersonal goal of eliciting help or better perceived support: people catastrophize to convey distress and elicit support. This study analyzed pain catastrophizing, attachment style and relational context regarding the perceived solicitous, distracting and punishing responses of significant others (SOs) to the patient’s pain in a sample of patients with advanced cancer. Gauthier et al. (2012) speculated that, if catastrophizing is a useful way to communicate pain and elicit desired support from significant others, it should be related to more frequent solicitous responses and thus to less frequent punishing responses. Consistent with their hypothesis, greater catastrophizing was related to more frequent perceived distracting and solicitous responses when adjusted for attachment style (Gauthier et al., 2012). However, there was no direct relationship between pain catastrophizing and punishing responses: this relationship was moderated by attachment anxiety and relationship to the SOs.

Forsythe et al. (2012) examined the role of perceived spouse responses to pain behavior and its association with attachment style, depression, disability, pain intensity, and pain behavior in a sample of CP patients. Their study highlighted that both perceived spouse responses to pain behaviors and attachment style were significant independent predictors of pain behavior and depression in pain patients. These results suggested that perceived negative partner responses may be associated with increased distress: attachment style may influence how patients perceive the responses of significant others, reporting higher levels of both pain behaviors and depressive symptoms if they perceive negative responses.

The studies of Forsythe et al. (2012) and Gauthier et al. (2012) both underlined the important role of the relational context in pain behavior. However, both also considered only the patients’ perception of others’ supportive responses, not the real reaction and support responses from significant others to patients’ pain behaviors.

Kowal et al. (2012) analyzed another factor that presupposes a reciprocal and interpersonal framework of CP: the self-perceived burden (SPB). SPB is another typical experience of pain patients that occurs when patients perceive that they are receiving more from significant others than they are giving them. The results underlined that SPB is not only significantly correlated with pain intensity, functional limitations, depressive symptoms, pain self-efficacy, and caregiver burden, but also with attachment anxiety (Kowal et al., 2012). In particular, CP patients with a high level of anxious attachment may be more demanding of their caregivers, and this may contribute to the caregivers’ burden as well as to the patients’ feelings of SPB.

Going further than previous studies, Monin et al. (2014) examined the interplay between partners’ attachment styles and indicators of individual (depressive symptoms) and relational (marital satisfaction) psychological health in older married couples where one partner had a musculoskeletal pain condition. They found that when one or both partners were insecurely attached, both reported greater depressive symptoms and lower marital satisfaction (Monin et al., 2014). In particular, if one partner’s had attachment anxiety, this was associated with greater depressive symptoms for both partners. What is more, spouses reported lower marital satisfaction when their CP partner had high anxious attachment. With regard to avoidant attachment, this was associated with lower marital satisfaction for both partners, whilst CP patients whose spouses had high avoidant attachment reported more depressive symptoms (Monin et al., 2014). Taken together, these results suggested that avoidant attachment impedes both the support-seeking and caregiving processes. To our knowledge, this is the first study that takes into account the role of the reciprocal influence of both partners’ attachment style in CP patients.

### The Future: Toward an Interpersonal Model of Pain

The current review suggests that the studies made after 2008, starting from the *Attachment Diathesis Model of Chronic Pain* (Meredith et al., 2008) investigated more deeply the psychological factors possibly involved in the relationship between attachment style and CP conditions. Moreover, very recent studies starting to move from the individual attachment pattern, originally proposed by the Meredith’s model (Meredith et al., 2008), toward a relational perspective. Indeed, starting from the hypothesis that insecure attachment style is associated with CP, in recent years researchers have first realized that this relationship is mediated by different psychological aspects, and then have finally stressed the interplay between partners’ attachment styles.

A CP condition is a source of great distress for both patients and their spouses or significant others, with a recent article (Pietromonaco et al., 2013) pointing out that attachment and dyadic processes can contribute to the health-related processes and outcomes. In a prototypical dyadic relationship, attachment style can influence the dyadic processes themselves. Each partner’s dyadic processes can influence and are influenced by physiological responses, affect, health behavior, and disease outcomes. With regard to CP, the dyadic features of the model include both the patients’ and partner’s reactions to CP (Pietromonaco et al., 2013). In particular, when insecurely attached patients react to pain by using interpersonal strategies, this leads to greater relational conflict that, in turn, may influence adjustment outcomes to pain (Pietromonaco et al., 2013). In line with the latter, Wilson and Ruben (2011) found that anxiously attached women respond more negatively to experimentally induced acute pain when with an anxiously attached partner. What is more, spouses with a high anxious attachment style reported a higher anxious mood than spouses with a low anxious attachment style (Porter et al., 2012). Indeed, patient pain represents a stressor not only for the patient but also for the partner. Spouses are involved in regulating negative affects in response to their partner’s pain, and thus spouses’ attachment is also important (Monin and Schulz, 2009; Monin et al., 2010).

In order to modify pain behaviors, future interventions should consider not only the attachment style of individuals with CP, but also that of their significant others. Indeed, an intervention strategy which is successful for one couple with a specific attachment pattern may not be effective for another with a different attachment pattern, and interventions should therefore be tailored accordingly.

## Conclusion

Integrating relationship context and attachment perspective may make it possible to expand and achieve a more elaborated model of how interpersonal patterns such as attachment style and interactions with significant others might influence adjustment and functioning in CP.

It will be important for future research to better investigate the “multicausal” mechanisms by which a partner’s attachment influences a patient’s psychological health in real-time support interactions, since a better understanding of these factors will help clinicians to elaborate more efficient and effective psychotherapy interventions.

## Author Contributions

AR, VT, GC, and LC have written the manuscript after a critically read of the target literature; in addition all the authors gave the final approval of the manuscript submitted for publication.

## Conflict of Interest Statement

The authors declare that the research was conducted in the absence of any commercial or financial relationships that could be construed as a potential conflict of interest.
